# Vitamin D Supplementation and Improvement in Insomnia Symptoms Among Malaysian Women With Vitamin D Deficiency: Protocol for a Randomized Controlled Trial

**DOI:** 10.2196/63804

**Published:** 2026-10-19

**Authors:** Rara Merinda Puspitasari, Zaswiza Mohamad Noor, Didik Setiawan, Norkasihan Ibrahim

**Affiliations:** 1 Faculty of Pharmacy and Health Sciences, Universiti Kuala Lumpur Royal College of Medicine Perak, No 3 Jalan Greentown, Ipoh, 30450, Malaysia, 60 175993403; 2 Department of Social and Administrative Pharmacy, Faculty of Pharmacy, Muhammadiyah University Purwokerto, Purwokerto, Indonesia; 3 Department of Pharmacy Practice and Clinical Pharmacy, Faculty of Pharmacy, Universiti Teknologi MARA, Selangor, Malaysia

**Keywords:** vitamin D, insomnia, sleep quality, actigraphy, randomized controlled trial, women, vitamin D deficiency, functional outcomes, sleep efficiency, Malaysian women

## Abstract

**Background:**

Insomnia is a prevalent sleep disorder associated with adverse physical, psychological, and functional outcomes. Emerging evidence suggests that vitamin D deficiency may contribute to sleep disturbances. Women are more likely to have lower serum vitamin D (25-hydroxyvitamin D [25(OH)D]) levels than men, and they also report a higher prevalence of insomnia. Although several studies have proposed that vitamin D supplementation may improve insomnia symptoms, findings remain inconsistent, and evidence from Southeast Asian populations is limited.

**Objective:**

This study aims to evaluate the effectiveness of weekly vitamin D₃ supplementation in improving insomnia symptoms, sleep quality, sleep efficiency, and functional outcomes among Malaysian women with vitamin D deficiency.

**Methods:**

This study is a double-blind, parallel-group, randomized placebo-controlled trial conducted among women aged 19‐50 years with serum 25(OH)D levels <20 ng/mL and insomnia symptoms. Participants are randomized to receive either vitamin D₃ capsules (50,000 IU/wk) or placebo (1 capsule/wk) for 9 weeks. The study is conducted in a community setting. Primary outcomes include insomnia severity assessed using the Insomnia Severity Index (ISI), sleep quality measured by the Pittsburgh Sleep Quality Index (PSQI), and sleep efficiency objectively assessed using actigraphy. Secondary outcomes include daytime sleepiness measured using the Epworth Sleepiness Scale (ESS) and functional outcomes assessed using the Functional Outcomes of Sleep Questionnaire (FOSQ-10). Assessments are conducted at baseline, midintervention, and postintervention.

**Results:**

Participant recruitment commenced in December 2023 and is expected to conclude in December 2026. As of July 2026, 42 participants had been screened, 33 eligible participants had been enrolled, 22 had completed the study procedures, 4 were in the intervention phase, and 1 had withdrawn due to loss of contact.

**Conclusions:**

If the efficacy of vitamin D3 supplementation in alleviating insomnia symptoms is demonstrated, it might be proposed as a supplementary approach to cognitive behavioral therapy (CBT) for insomnia. Screening for vitamin D deficiency may also be considered in individuals presenting with insomnia symptoms prior to initiating pharmacological treatment.

## Introduction

### Background

Insomnia, one of the most prevalent sleep disorders, is characterized by dissatisfaction with both sleep quantity and quality. Individuals with insomnia commonly experience difficulty initiating and maintaining sleep, frequent nocturnal awakenings, and early-morning awakenings with an inability to return to sleep, often resulting in daytime fatigue and impaired functioning [1,2]. Chronic insomnia is associated with multiple adverse health outcomes, including reduced quality of life and an increased risk of chronic conditions such as cardiovascular disease, musculoskeletal disorders, diabetes, and cognitive decline, as well as heightened physical and psychological morbidity. In addition, sleep deprivation has been linked to altered immune function [3-7].

Globally, the prevalence of insomnia varies between 10% and 60%, depending on population characteristics and assessment criteria [8-10]. In Malaysia, the prevalence of insomnia is also notable. A community-based survey conducted in 4 urban regions reported that 33.8% of adults experienced insomnia symptoms, while 12.2% had chronic insomnia [11]. Numerous studies examining sex differences in sleep disorders have consistently shown that women are more likely to experience insomnia symptoms than men [12-14]. Despite this high prevalence, insomnia among Malaysian women remains an underresearched area, especially in relation to potential nutritional or physiological determinants.

Vitamin D deficiency (VDD) is another common yet often overlooked health issue in Malaysia. Although the country receives abundant sunlight throughout the year, several studies have reported a high prevalence of VDD among certain populations, particularly women, due to indoor lifestyles, cultural clothing practices, and limited dietary intake of vitamin D. Furthermore, natural food sources rich in vitamin D, such as oily fish, fish liver oil, and wild mushrooms, are not widely available or commonly consumed in many low- and middle-income countries, contributing to this deficiency [15].

Beyond its established role in bone metabolism, vitamin D has been increasingly recognized for its involvement in neurological and endocrine pathways related to sleep regulation and circadian rhythm. It is suggested that vitamin D plays a significant role in serotonin and melatonin regulation [16,17]. The active form of vitamin D, 1,25-dihydroxyvitamin D₃ (1,25(OH)₂D₃), binds to vitamin D receptors (VDRs) that are widely distributed in several brain regions responsible for sleep regulation, including the hypothalamus, prefrontal cortex, and substantia nigra [15,18]. Vitamin D regulates the expression of tryptophan hydroxylase 2 (TPH2), the rate-limiting enzyme in the conversion of tryptophan to serotonin in the brain. Adequate serotonin levels are essential for mood stability, and serotonin acts as a precursor for melatonin synthesis in the pineal gland via N-acetyltransferase and hydroxyindole-O-methyltransferase (HIOMT) enzymatic pathways [17,18]. Consequently, low vitamin D status may lead to impaired serotonin production and disrupted melatonin rhythms, contributing to poor sleep quality and insomnia symptoms [19]. This mechanistic pathway provides a plausible explanation for the observed association between VDD and altered circadian rhythm and supports the rationale for evaluating vitamin D₃ supplementation as a potential therapeutic strategy for insomnia.

Emerging evidence suggests that insufficient vitamin D levels may disrupt circadian rhythms and sleep architecture, contributing to sleep disturbances such as insomnia. A meta-analysis reported that VDD (defined as serum 25-hydroxyvitamin D [25(OH)D] levels <20 ng/mL) was associated with an increased risk of sleep disorders (odds ratio [OR] 1.50, 95% CI 1.31‐1.72) [19]. In addition, women may be particularly susceptible to insomnia associated with VDD. A study involving 459 women revealed a significant relationship between the circadian rhythm of sleep and dietary vitamin D intake, showing that a later sleep acrophase—the time at which the peak of rhythm occurs—was associated with higher dietary vitamin D intake [20]. Despite growing global evidence linking VDD with poor sleep quality, local data examining this relationship among Malaysian women, a population at higher risk for both conditions, remain limited.

### Previous Research on Vitamin D Supplementation for Insomnia

Several studies have investigated the effects of vitamin D supplementation on sleep outcomes; however, findings remain inconsistent, largely due to methodological variability. Majid et al [21] reported that individuals aged 20‐50 years with sleep disorders who received 50,000 IU of vitamin D3 weekly for 8 weeks experienced significant improvements in Pittsburgh Sleep Quality Index (PSQI) scores compared with the placebo group (PG; *P*<.05). Conversely, Mason et al [22] found that daily supplementation of 2000 IU vitamin D₃ in overweight postmenopausal women did not significantly improve sleep quality or quality of life compared to the placebo (*P*>.05). The differences in study population characteristics, dosage, and intervention duration may account for these inconsistent findings.

Another limitation in the existing studies is the lack of objective sleep measures that provide quantifiable and reliable data on sleep efficiency, latency, and duration. Most studies relied exclusively on subjective self-assessment tools, such as the PSQI or the Insomnia Severity Index (ISI), which may be influenced by recall bias and perceptual differences [15,19]. Additionally, the inclusion of participants with comorbidities (eg, fibromyalgia, depression, or obstructive sleep apnea) and varying vitamin D status complicates the interpretation of outcomes, as these conditions independently affect sleep quality [23-25].

To address these gaps, the present study was designed to incorporate both subjective (validated questionnaires) and objective (actigraphy-based) sleep assessments, focusing on a homogeneous group of Malaysian women with VDD. This approach is intended to yield more robust evidence on the effect of vitamin D supplementation in improving insomnia outcomes within a clearly defined population. By integrating subjective and objective sleep measures, this study aims to offer contextually relevant insights into the potential role of vitamin D₃ supplementation as a nonpharmacological strategy for improving sleep health among Malaysian women with VDD.

### Aims and Objectives

This study aims to evaluate the effectiveness of weekly vitamin D₃ supplementation (50,000 IU) in improving insomnia symptoms among women with VDD. The primary objective is to determine the effect of vitamin D₃ supplementation on insomnia-related parameters, including insomnia severity assessed using the ISI, sleep quality measured by the PSQI, and sleep efficiency, which is defined as the ratio of total sleep time to time spent in bed and is objectively assessed using actigraphy.

The secondary objectives are to evaluate the broader effects of vitamin D₃ supplementation on daytime sleepiness, measured using the Epworth Sleepiness Scale (ESS), and on functional outcomes and sleep-related quality of life, assessed using the Functional Outcomes of Sleep Questionnaire (FOSQ-10). Sleep efficiency is considered an important indicator of restorative sleep quality, while functional outcomes reflect the impact of sleep disturbances on daily functioning, productivity, vigilance, and social engagement.

We hypothesize that vitamin D₃ supplementation will significantly improve insomnia symptoms, as indicated by enhanced sleep efficiency and reduced ISI and PSQI scores, compared with placebo. We further hypothesize that vitamin D₃ supplementation will improve daytime alertness and functional quality of life compared with baseline and the PG.

## Methods

### Trial Design

This study is designed as a double-blind, parallel-group, randomized, placebo-controlled clinical trial to evaluate the effectiveness of vitamin D₃ supplementation (50,000 IU) for insomnia symptoms among women with VDD. The study protocol serial number is VDSI01, and the trial is registered with the International Standard Randomized Controlled Trial Number (ISRCTN) registry (ISRCTN69678101). The trial will be conducted in community settings in Ipoh, Perak, Malaysia, and will follow the SPIRIT (Standard Protocol Items: Recommendations for Interventional Trials) 2025 (Checklist 1) guidelines for clinical trial protocols [26].

### Study Participants and Eligibility Criteria

Women aged 19‐50 years who reside in Ipoh, Perak, Malaysia, will be screened for eligibility. Participants will be eligible for inclusion if they have a serum 25(OH)D level <20 ng/mL (50 nmol/L) and report insomnia symptoms, defined as a PSQI score ≥5, are not currently taking vitamin D supplements or supplements containing vitamin D in the past 3 months, and are nonsmokers and nonalcohol users.

Participants will be excluded if they are pregnant or lactating, share a sleeping environment with an infant younger than 2 years, or have chronic medical conditions such as diabetes mellitus, cardiovascular diseases, thyroid disorders, osteoporosis, malignancy, malabsorption syndromes, or psychiatric disorders.

Individuals taking medications that could interfere with sleep outcomes or vitamin D metabolism will also be excluded. These include corticosteroids, anticonvulsants, cytotoxic agents, antiretroviral drugs, narcotics, antidepressants, and antipsychotics. Participants using antihistamines, particularly first-generation agents (eg, diphenhydramine and chlorpheniramine), will be excluded due to their sedative and anticholinergic properties, which may alter sleep latency, total sleep time, and sleep efficiency. These pharmacological effects could confound both subjective and objective sleep assessments.

### Sampling Method and Recruitment

#### Sample Size

The sample size was determined for a parallel randomized controlled trial with a superiority design, where the primary outcome (sleep quality score) is a continuous variable. The calculation was performed using the Sealed Envelope (Sealed Envelope Ltd) online sample size calculator based on standard formulas for comparing 2 independent means [27].

The following assumptions were used: significance level (α)=.05 (2-sided); statistical power (1 − β)=80%; allocation ratio (vitamin D3:placebo)=1:1; expected mean difference (d)=1.92≈2 in sleep quality score between groups [21]; SD 3.14 [21]. The expected mean difference and SD were derived from previous studies evaluating the effects of vitamin D supplementation on sleep outcomes.

Based on these parameters, the minimum required sample size was 38 participants per group. To account for potential attrition during the study period, a 10% dropout rate was applied [28]. This estimate was considered appropriate given the relatively short intervention duration (9 wk), the low-risk nature of vitamin D supplementation, and the use of regular follow-up reminders to enhance adherence. Therefore, the final sample size was determined to be 42 participants per group, resulting in a total sample size of 84 participants.

#### Sampling and Recruitment Process

Convenience sampling will be used to recruit participants from the community or through community-based recruitment. Recruitment will be conducted through the dissemination of study advertisements via posters and leaflets distributed in public areas, pharmacies, and educational institutions, as well as through social media platforms. The advertisements will provide brief information about the study objectives, eligibility criteria, and benefits of participation, including free serum vitamin D level assessment and a token of appreciation.

Interested individuals will contact the research team using the contact details provided in the advertisements. An appointment will then be scheduled for an initial screening session, during which the investigator will explain the study procedures and objectives in detail. Eligible participants will receive both verbal and written information about the study, including an information sheet outlining the study’s purpose, procedures, potential benefits, and participants’ rights. Written informed consent will be obtained prior to enrollment, with emphasis on the voluntary nature of participation, the right to withdraw at any time without penalty, and assurances of confidentiality. Upon completion of the study, participants will receive a token of appreciation in the form of cash.

Participant recruitment and follow-up will be conducted in parallel, with participants enrolled in overlapping cohorts. The study is supported by the availability of 2 actigraphy devices, allowing simultaneous assessment of participants.

#### Assessment for Eligibility

A series of evaluations and tests will be conducted to determine the participants’ eligibility for the study. These data will also be included as baseline data for participants.

The screening process will include:

1. Evaluation of participants’ characteristics based on the inclusion and exclusion criteria.
2. Assessment of insomnia symptoms using validated questionnaires (ISI and PSQI).
3. Measurement of serum 25(OH)D levels in the blood. The participants must have a low baseline serum 25(OH)D level (<20 ng/L).

Those who meet all the criteria will be enrolled in the study.

### Blinding and Randomization

Eligible participants were randomly assigned in a 1:1 ratio to either the intervention group (IG) or the PG using a block randomization method (block size =4) generated by a computer-based randomization tool. Block randomization was used to ensure a balanced allocation of participants to each group, particularly given the anticipated recruitment rate. A block size of 4 was selected based on the 2 intervention arms (vitamin D₃ and placebo).

Allocation concealment will be ensured using sequentially numbered, opaque, sealed envelopes (SNOSE) prepared by an independent third party (an appointed pharmacist) who is not involved in participant recruitment or outcome assessment. Each envelope will contain the group assignment (labelled as group A or B) according to the computer-generated randomization sequence and will be opened only after participant enrollment. The allocation list will be securely maintained by the third party in a locked cabinet, and investigators will not have access to the sequence throughout the study period.

To maintain blinding, the intervention and placebo will be labelled as group A or B, and the identity of each group (IG or PG) will remain concealed until completion of data analysis. Both participants and investigators will remain blinded to group assignments throughout the study period. Participants will not be informed of their postintervention serum 25(OH)D results until completion of the study to minimize the risk of unblinding due to perceived treatment effects. Researchers will have access to laboratory results for outcome assessment; however, group allocation will remain concealed throughout the study. To monitor blinding integrity, any instances of suspected unblinding—such as those arising from perceived treatment effects or adverse events—will be documented during the study and considered during data interpretation.

A detailed participant flow diagram (Figure 1) illustrates the processes of recruitment, screening, randomization, follow-up, and analysis and is consistent with the CONSORT (Consolidated Standards of Reporting Trials) 2010 guidelines.

**Figure 1.**
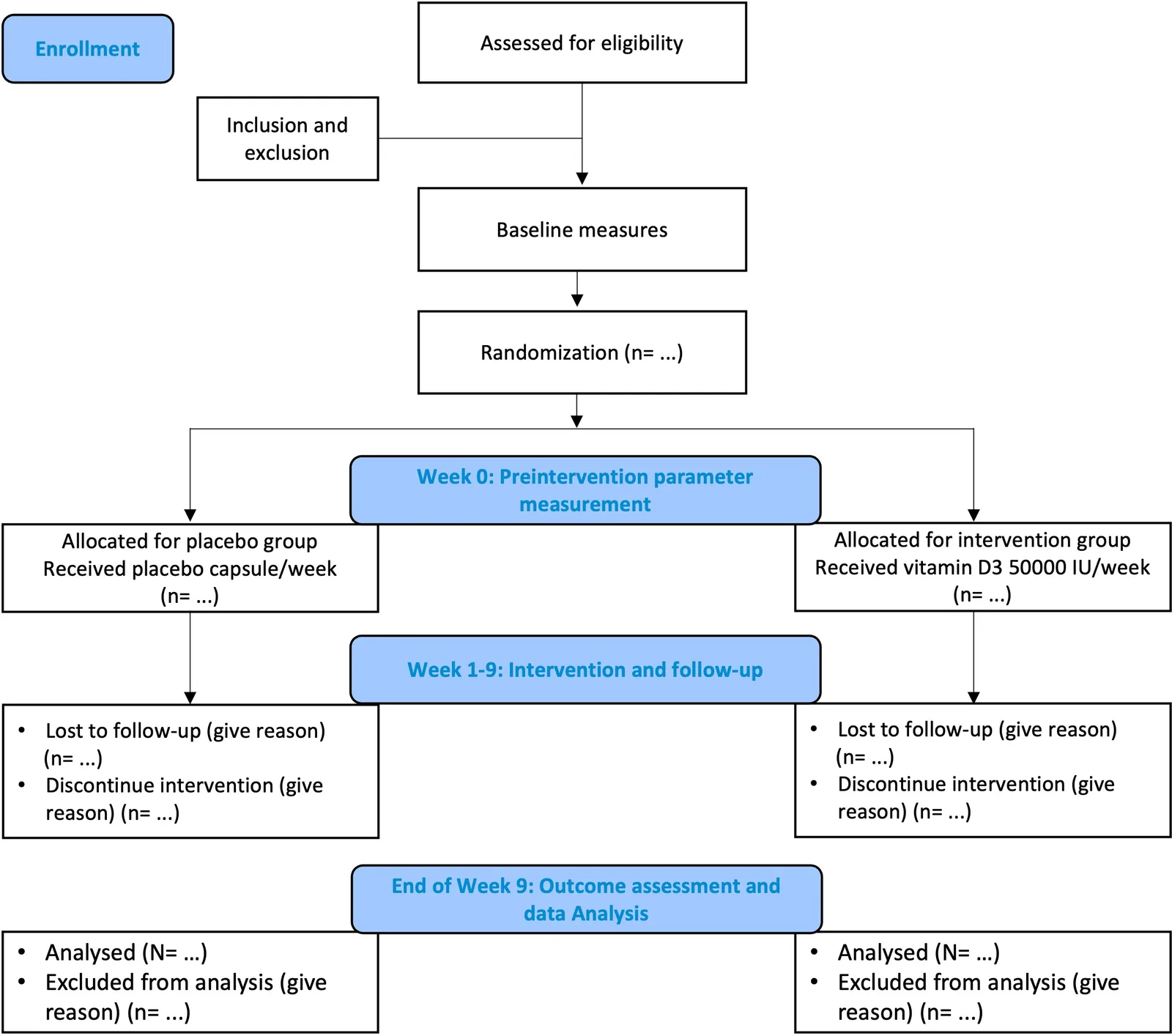
CONSORT (Consolidated Standards of Reporting Trials) study diagram. The diagram follows CONSORT 2010 guidance adapted for protocol reporting. Numbers will be completed upon study completion.

### Intervention

The intervention is initiated after completion of the 7-day baseline actigraphy recording and continued for 9 weeks. Participants in the IG will receive vitamin D₃ (cholecalciferol) capsules at a dose of 50,000 IU weekly for 9 consecutive weeks. The PG will receive capsules containing inert excipients, administered at the same frequency. Participants will be instructed to consume the capsules after breakfast on the same day each week.

Figure 1 displays the details of the study flow. Baseline parameters will be taken at recruitment, and participants will be randomly assigned into 2 arms. Outcome assessments will be conducted at week-0 and week-9. Participants will be instructed to maintain their normal levels of physical activity, keep their usual diet, refrain from taking any additional dietary supplements, and refrain from attempting to lose weight during the study period.

### Outcome Measures

An overview of the study assessments and measurement time points is presented in Table 1.

**Table 1.** Summary of measurements.

Measurements	Instruments	Time frame		
		T0^a^	T1^b^	T2^c^
Sociodemographic and lifestyle	Questionnaire	✓	—^d^	—
Serum 25(OH)D^e^ level	Biochemistry test	✓	—	✓
Sleep quality	PSQI^k^	✓	—	✓
Sleepiness severity	ISI^g^	✓	—	✓
Subjective daytime sleepiness	ESS^h^	—	✓	✓
Daytime function	FOSQ-10^i^	—	✓	✓
Sleep parameter assessment	Actigraph	—	✓	✓

a Screening phase.

b Week-0: baseline assessment.

c Week-9: end of study week.

d Not applicable.

e 25(OH)D: 25-hydroxyvitamin D.

f PSQI: Pittsburgh Sleep Quality Index.

g ISI: Insomnia Severity Index.

h ESS: Epworth Sleepiness Scale.

i FSOQ-10: Functional Outcomes of Sleep Questionnaire-10.

#### Vitamin D Status

Blood samples will be collected at BP Specialist Center, Ipoh, Perak, Malaysia, in the early morning following an 8-hour overnight fast at baseline (week 0) and postintervention (week 9). Serum 25(OH)D levels will be measured using a chemiluminescent microparticle immunoassay (CMIA). Only participants with baseline serum 25(OH)D levels less than 20 ng/mL will be included in the study.

#### Primary Outcomes

##### Insomnia Severity

Insomnia severity will be assessed using the ISI, a validated 7-item self-reported questionnaire evaluating difficulties with sleep initiation, sleep maintenance, early-morning awakening, and the impact of sleep problems on daily functioning. Each item is rated on a 5-point Likert scale (0‐4), with total scores ranging from 0 to 28. Higher scores indicate more severe insomnia, and a reduction in total score reflects improvement.

##### Sleep Quality

Sleep quality will be evaluated using the PSQI, which assesses seven domains of sleep, including subjective sleep quality, sleep latency, sleeping duration, habitual sleep efficiency, sleep disturbances, daytime dysfunction, and use of sleep medication. The global PSQI score ranges from 0 to 21, with scores >5 indicating poor sleep quality.

##### Sleep Efficiency

Sleep efficiency was defined as the ratio of total sleep time to time spent in bed. Sleep-wake patterns were assessed using the ActiTrust (Condor Instruments) device, worn continuously for 7 consecutive days at baseline and postintervention. Actigraphy data were recorded continuously and analyzed using the device’s validated proprietary algorithm with standard epoch settings. Participants were instructed to press the event marker button at bedtime and upon final awakening, which was used to define rest intervals in place of sleep diaries.

### Secondary Outcomes

#### Daytime Sleepiness

Subjective daytime sleepiness will be measured using the ESS. The ESS consists of 8 items assessing the likelihood of dozing in common daily situations, rated on a 4-point Likert scale (0‐3). Total scores range from 0 to 24, with scores of ≥10 indicating excessive daytime sleepiness.

#### Functional Outcomes

Daytime functioning and sleep-related quality of life will be assessed using the FOSQ-10. This instrument evaluates the impact of sleepiness on daily activities across 5 domains: general productivity, activity level, vigilance, social outcomes, and intimacy. Higher total scores indicate better functional status.

### Data Collection Procedures

Assessments will be conducted at baseline (week 0), midintervention (week 5), and postintervention (week 9).

#### Preintervention Assessment (Week 0)

Following randomization, participants will complete preintervention assessments involving subjective measurements using structured questionnaires (ESS and FOSQ-10) and objective measurements of sleep parameters using an Actigraph for 1 week during all sleep periods (only during the first week or week 0). These data will serve as baseline information for the study participants, in addition to data collected during screening before enrollment (vitamin D level, ISI score, and PSQI score).

#### Intervention (Weeks 1-9)

Participants in the IG will be instructed to consume 50,000 IU of vitamin D3 capsules, 1 capsule per week over 9 weeks, whereas those in the PG will take a placebo capsule, 1 capsule per week, for the same duration. Participants will be instructed to take the product once weekly with food in the morning. Participants will be asked to report any drug use and any changes in their health condition that occur throughout the trial period.

#### Postintervention Assessment (End of Week 9)

Postintervention outcome assessments will be conducted after 9 weeks, involving 7 days (1 wk) of actigraphy measurements and the collection of questionnaire scores at the end of the intervention week (PSQI, ISI, ESS, and FOSQ-10 scores). A vitamin D level test will also be carried out at the end of the intervention week. Data analysis will then be carried out to obtain the study results.

#### Strategies to Ensure Adherence to the Intervention

The researcher will actively contact participants via message or phone call to remind them of their vitamin D or placebo intake and to ensure they take the dose every week. A recurring weekly reminder will also be set on the participants’ phones, scheduled for the same morning each week over a 9-week period. Participants will be asked to report any missed doses, side effects, or changes in health status throughout the study. Participants who miss a dose for 1 week or cannot be reached for follow-up will be withdrawn from the study.

Participants may be withdrawn from the study if they voluntarily withdraw consent, are lost to follow-up, discontinue the intervention, or develop medical conditions during the study period that may interfere with participation or outcome assessment.

### Feasibility and Acceptability

Feasibility will be assessed based on recruitment rate, participant retention, and adherence to supplementation. Acceptability will be evaluated through participant feedback on intervention tolerability and study procedures collected at the end of the intervention period.

### Safety Monitoring

Participants will be monitored for adverse events throughout the study period. Any reported adverse events will be documented and reviewed by the study team. Participants experiencing clinically significant adverse effects will be referred for appropriate medical evaluation. Parameters that may indicate the need for study discontinuation will be assessed, including a serum 25(OH)D level higher than 150 ng/mL (375 nmol/l), a serum calcium level higher than 10.3 mg/dL, hypercalciuria, and a glomerular filtration rate less than 50 mL/min/1.73 m^2^ [29]. The participant will receive the necessary treatment accordingly.

### Statistical Analysis

The SPSS (version 23; IBM Corp) Statistics for Windows program will be used to analyze all data. Prior to selecting the appropriate SPSS test for analysis, the data will be assessed for normality and homogeneity. Normality will be examined using the Kolmogorov–Smirnov test and a Q-Q plot. Descriptive statistics will be used to analyze categorical variables, such as participant characteristics. Quantitative data will be expressed as mean (SD), while qualitative data will be presented as numbers and percentages. The primary analysis will evaluate the effect of vitamin D supplementation on insomnia symptoms, determined by comparing the change from baseline to postintervention (week 9) between the intervention and PGs. A *P* value <.05 will be considered significant.

The statistical analyses will be performed as follows:

1. Comparisons between the characteristics of participants in the IG and the PG will be examined using a 2-tailed Mann-Whitney *U* test.
2. The comparison between outcomes before and after the intervention within the group will be examined using the Wilcoxon signed-rank test.
3. The comparison of postintervention outcomes between A and B will be examined using the Mann-Whitney *U* test.
4. Where appropriate, analysis of covariance (ANCOVA) will be performed to adjust for predefined covariates (eg, age, BMI, baseline values).

### Ethical Considerations

The trial adhered to the ethical standards of the Declaration of Helsinki. This study protocol has been examined and approved by the Universiti Kuala Lumpur Research Ethics Committee (UREC), with the approval number (reference number: UNKIL REC/2022/0017). Any protocol amendments will be submitted for approval to the Universiti Kuala Lumpur Research Ethics Committee and will be updated in the trial registry prior to implementation.

All participants provided written informed consent before enrollment. All information and data obtained during this study will be kept confidential. Participants will not have access to their personal information or study data. All hard copy data collected from participants will be securely stored and will be accessible only to the researchers. Additionally, all information and softcopy data will be saved on the principal investigator’s password-protected laptop (1 laptop only). The data are stored on a password-protected laptop integrated with secure cloud storage, with access restricted to the investigator’s account only.

Participants’ names will be converted to codes or study IDs (eg, P01 and P02) and recorded on the collection form. The soft copy of the database will be password-protected, with only the principal investigator and coinvestigator having access. After the study is completed, all data, both the hard copy and soft copy files, will be retained for study purposes for 5 years before being permanently discarded from the database. Individual patient records will not be shared with anyone outside the research team.

## Results

This study was partially funded by the Short-Term Research Grant from Universiti Kuala Lumpur Royal College of Medicine Perak. Participant recruitment commenced in December 2023 and is expected to conclude by December 2026, subject to recruitment progress and completion of follow-up assessments. As of July 2026, 42 participants had been screened, 33 eligible participants had been enrolled, 22 had completed the study procedures, 4 were in the intervention phase, and 1 had withdrawn due to loss of contact. Data collection is ongoing, and data analysis has not yet commenced. The findings are expected to be disseminated through peer-reviewed publications and academic conference presentations upon the completion of data collection and analysis. Figure 1 illustrates the study processes consistent with CONSORT 2010 guidelines.

## Discussion

### Potential Role of Vitamin D in Sleep and Insomnia

VDRs and the enzyme 1α-hydroxylase, which converts vitamin D to its active form, 1,25(OH)₂D₃, are expressed in brain regions involved in sleep–wake regulation, cognition, and mood, including the hypothalamus, hippocampus, prefrontal cortex, and substantia nigra [15,30]. Through these pathways, vitamin D₃ modulates the synthesis of serotonin and dopamine, neurotransmitters essential for circadian rhythm stability, emotional regulation, and cognitive performance [17]. Adequate vitamin D3 levels may therefore improve sleep quality and circadian alignment, reducing daytime fatigue and enhancing functional outcomes such as alertness, productivity, and social functioning [31-33]. In addition, vitamin D₃ has anti-inflammatory and neuroprotective properties that may reduce cytokine-mediated neural stress and oxidative burden associated with sleep disturbances and daytime somnolence [34,35]. By promoting neuromuscular efficiency and stabilizing neurotransmission, adequate vitamin D3 levels may further support psychomotor performance and general vitality during waking hours. The influence of vitamin D3 extends beyond sleep regulation to encompass broader neurocognitive and physiological processes that contribute to daytime functioning.

However, VDD is prevalent among individuals seeking sleep medication for insomnia and may contribute to common sleep-related symptoms, including chronic nonspecific pain, poor sleep quality, and impaired daytime functioning [15]. Several studies have suggested that changes in vitamin D metabolism play an important role in the severity and clinical presentation of sleep disorders. Low serum 25(OH)D levels have been independently associated with sleep maintenance difficulties (OR 0.84) [20]. A study by Zhao et al [36] proposed that measuring serum 25(OH)D levels may be useful in predicting pharmacotherapy treatment outcomes in individuals with chronic insomnia. It has been reported that a serum 25(OH)D concentration <20 ng/ml in individuals with chronic insomnia was independently associated with a higher probability of treatment nonresponse at 2 months (OR 11.636, 95% CI 3.966-34.142; *P*<.001). Evidence also suggests potential benefits of vitamin D3 supplementation for sleep-related outcomes. A study involving 940 healthy adolescent girls who received 9 weeks of vitamin D3 supplementation (50,000 IU vitamin D3/wk) demonstrated a significant improvement in cognitive performance (*P*<.001) and a reduction in the occurrence of insomnia and daytime sleepiness. The prevalence of insomnia decreased from 15.0% to 11.3% and sleepiness decreased from 15.6% to 14.7%. Additionally, cases with both insomnia and sleepiness decreased from 8.0% to 6.1% (*P*<.05) [28]. Improved sleep quality resulting from vitamin D sufficiency may restore circadian alignment and reduce daytime fatigue, thereby enhancing functional outcomes such as alertness, productivity, and social engagement. This theoretical framework supports the inclusion of the ESS and the FOSQ-10 in this study to capture the broader impact of sleep improvement on daily activities and quality of life.

However, findings from vitamin D supplementation trials remain inconsistent. One randomized controlled trial failed to demonstrate any significant effect of vitamin D supplementation on various sleep outcomes in a vitamin D-deficient population. Among 189 participants, 92 received a bolus dosage of vitamin D 100,000 IU (2500 mg), followed by a weekly dose of 20,000 IU (500 mg), whereas 97 were given a placebo. The result showed that vitamin D supplementation did not have a significant effect on mean sleep duration, mean daytime sleepiness score, or recovery from inadequate sleep duration, excessive daytime sleepiness, or insomnia after 4 months of treatment [37]. Similarly, Mason et al [22] found that daily supplementation with 2000 IU of vitamin D₃ did not improve sleep quality or quality of life among overweight postmenopausal women. Compared to the placebo, vitamin D did not improve quality of life (*P*>.05) or sleep (*P*=.21) in women. These discrepancies may be attributed to differences in study populations, baseline vitamin D status, dosing regimens, supplementation duration, and reliance on subjective sleep measures.

The inconsistent findings from previous studies underscore the need for more randomized controlled trials incorporating clearly defined populations, standardized dosing protocols, and both objective and subjective sleep assessments. This study protocol outlines the design and rationale for a randomized controlled trial investigating the potential benefits of vitamin D3 supplementation in improving insomnia symptoms among Malaysian women with VDD. The study design holds promise for addressing the underexplored association between vitamin D status and sleep quality within this specific population. The decision to focus on Malaysian women with VDD is strategic, given the higher prevalence of VDD and insomnia in this population. The study aims to provide insights into whether addressing VDD through supplementation can lead to improvements in insomnia symptoms, a significant concern for women’s health and well-being.

While the present study offers valuable insights into the effect of vitamin D₃ supplementation on insomnia symptoms, several limitations should be acknowledged. The recruitment of participants from a single geographical location (Ipoh, Perak) and the use of strict inclusion and exclusion criteria may restrict the generalizability of the findings to broader populations of women with insomnia, particularly those with comorbid conditions or from different sociodemographic backgrounds. This decision was intentional to enhance internal validity and minimize confounding factors. It strengthens causal inference by ensuring that the observed effects can be attributed specifically to vitamin D₃ supplementation, rather than to comorbid conditions or medications affecting sleep or vitamin D metabolism. Future studies should consider multicenter recruitment, broader inclusion criteria, and more diverse populations to enhance external validity and generalizability of the findings.

This study also benefits from its placebo-controlled design and the integration of both subjective and objective sleep measures, allowing comprehensive evaluation of insomnia-related outcomes. The findings are expected to contribute meaningful evidence to the growing literature on vitamin D and sleep health and may inform future nonpharmacological interventions for insomnia among individuals with VDD. Additionally, the findings may pave the way for targeted therapies addressing both VDD and sleep problems in this population. The findings of this study may be generalizable to adult women with VDD in similar community settings. However, caution should be exercised when extrapolating results to other populations, including men, older adults, or individuals with different clinical conditions.

### Conclusions

This current study builds upon previous research that has demonstrated the positive effects of vitamin D supplementation on insomnia symptoms. This study aims to provide preliminary evidence on the role of vitamin D3 supplementation in improving insomnia symptoms among Malaysian women with VDD. It focuses on a different population and measures more insomnia-related outcomes, both subjectively and objectively, to provide more reliable findings. Ultimately, this randomized controlled trial will contribute valuable evidence supporting the potential benefits of vitamin D supplementation in improving insomnia symptoms among individuals with VDD, especially Malaysian women. The findings are expected to contribute to a better understanding of the relationship between vitamin D status and sleep and to inform future, larger randomized controlled trials. In addition, it will emphasize the importance of considering vitamin D status in the management of sleep disturbances and advocate for further research to optimize interventions for diverse populations.

## Supplementary material

10.2196/63804 Checklist 1 SPIRIT checklist.

## Abbreviations

25(OH)D: 25-hydroxyvitamin D
ANCOVA: analysis of covariance
CBT: cognitive behavioral therapy
CMIA: chemiluminescent microparticle immunoassay
CONSORT: Consolidated Standards of Reporting Trials
ESS: Epworth Sleepiness Scale
FSOQ-10: Functional Outcomes of Sleep Questionnaire-10
HIOMT: hydroxyindole-O-methyltransferase
IG: intervention group
ISI: Insomnia Severity Index
ISRCTN: International Standard Randomized Controlled Trial Number
OR: odds ratio
PG: placebo group
PSQI: Pittsburgh Sleep Quality Index
RCT: randomized controlled trial
SNOSE: sequentially numbered, opaque, sealed envelopes
SPIRIT: Standard Protocol Items: Recommendations for Interventional Trials
TPH2: tryptophan hydroxylase 2
UREC: Universiti Kuala Lumpur Research Ethics Committee
VDD: vitamin D deficiency
VDI: vitamin D insufficiency
VDR: vitamin D receptor
